# The Circadian Rhythm of Asthma Immune Metabolism

**DOI:** 10.1155/jimr/3246881

**Published:** 2025-12-28

**Authors:** Manchen Lu, Feiyan Cheng, Xiaoman Zhao, Zhuiyue Wang, Jing Chen, Yuanyuan Zhang, Shengyuan Huang, Xinguang Zhang, Yazun Liu, Wenbo Yao, Zheng Xue

**Affiliations:** ^1^ Shanghai Municipal Hospital of Traditional Chinese Medicine, Shanghai University of Traditional Chinese Medicine, Shanghai, China, shutcm.edu.cn; ^2^ First Clinical College of Shanxi Medicine University, Shanxi University of Chinese Medicine, Jinzhong, China, sxtcm.com; ^3^ Key Laboratory of Organ Regeneration and Transplantation of Ministry of Education, Institute of Immunology, The First Hospital, Jilin University, Changchun, China, jlu.edu.cn

**Keywords:** asthma, circadian rhythm, immune metabolism, natural medicines, therapeutic strategies

## Abstract

Asthma is a chronic inflammatory respiratory disease with a distinct circadian rhythm. Common symptoms like cough, wheezing, and dyspnea, are worse at night and in the early morning (around 4 a.m.). The circadian pattern of asthma is intrinsically connected to the body’s internal clock, which regulates a wide range of biological processes via transcription‐translation feedback loop (TTFL). BMAL1 and CLOCK play a central role in the regulatory networks that control metabolism and immune function. When circadian rhythms are disrupted, it can result in metabolic disorders, immune dysfunction, and a worsening of asthma symptoms. A deeper understanding of the mechanisms underlying circadian rhythms may pave the way for innovative asthma treatments. Furthermore, the exploration and application of natural drugs and targeted therapies focusing on circadian rhythm genes show potential as a key approach for future precision treatment of asthma.

## 1. Introduction

### 1.1. Definition and Epidemiology of Asthma

Asthma is an inflammatory condition impacting the airways with clinical manifestations of increased airway hyperreactivity and reversible airflow obstruction. As a chronic airway condition, asthma poses a significant worldwide health challenge. It impacts individuals across all age groups and, if not properly managed, can impose considerable restrictions on daily activities and even prove fatal. Notably, the incidence of asthma is on the rise globally, with a particularly pronounced increase observed in child populations [1, 2]. Asthma manifests with a diverse array of symptoms, including wheezing, shortness of breath, chest tightness, and cough, which tend to be more pronounced at night and during the early morning [1, 3]. These episodes are typically associated with widespread but variable airflow obstruction within the lungs, which is often reversible spontaneously or with treatment [4]. Asthma is considered a chronic condition. However, it may also be considered acute when symptoms temporarily worsen, such as during an asthma attack. An acute asthma exacerbation is a sudden, severe worsening of asthma symptoms. During an acute exacerbation, airway inflammation and obstruction rapidly increase, making breathing increasingly difficult. Conditions can worsen within minutes or persist for hours, sometimes even days [5]. Chronic asthma symptoms are usually milder than acute asthma attacks. Chronic asthma symptoms can also affect a person’s quality of life, fluctuate in severity, and are easily affected by environmental triggers. Unlike acute asthma attacks, chronic asthma symptoms are characterized by persistent airway inflammation and a persistently hyperresponsive immune system, rather than a sudden escalation of symptoms [6, 7].

Asthma endotypes can be broadly regarded as Type 2 (T2) high (eosinophilic), T2‐low or non‐T2 (noneosinophilic) [8–10]. The most common type is T2‐high asthma, in which allergens induce eosinophils as the primary inflammatory effector cells [11–15]. The diagnosis of T2‐high asthma relies on the well‐established criterion of elevated eosinophil counts in peripheral blood or sputum, which are assessed using standardized thresholds. In contrast, T2‐low asthma lacks a universally accepted definition, is characterized by the absence of T2‐high inflammation, and currently has no defining biomarker. Therefore, seeking accurate phenotypic classification poses a major challenge to the medical research community [16, 17].

Over the past two decades, asthma has escalated into a global health issue affecting over 260 million people, with rising prevalence, morbidity, and mortality worldwide [18, 19]. Key environmental and occupational factors contribute to this burden; air pollution is a pivotal risk for exacerbating and progressing the disease in adults [20], while occupational exposures account for over 25% of asthma cases among the working‐age population [21]. In addition to common external triggers, diseases such as gastroesophageal reflux disease (GERD), laryngopharyngeal reflux (LPR), vocal cord dysfunction (VCD), obstructive sleep apnea, and unhealthy lifestyle habits may exacerbate the severity of asthma or change its manifestations [22–29]. These factors may have a complex impact on asthma by affecting airway inflammation, triggering gastric acid reflux, causing respiratory restriction, or affecting mood.

### 1.2. Circadian Rhythms Influence Respiratory Homeostasis

Circadian rhythms are self‐sustaining in the absence of environmental cues, maintained by an intracellular transcription‐translation feedback loop (TTFL) with a period of ~24 h. Two transcription factors, circadian rhythmic motor output cycle kaput (CLOCK) and brain and muscle ARNT‐like 1 (BMAL1), bind to the E‐box DNA motif of the circadian clock control gene (CCG), and then recruit coactivators CBP/p300 (a group of histone acetyltransferases), as well as thyroid hormone receptor‐associated protein 3 (THRAP3), steroid receptor coactivator 2 (SRC‐2), and several other peptides. These then activate cycle genes (PER1, PER2, and PER3) and cryptochrome genes (CRY1 and CRY2) in the primary feedback loop, and REV‐ERB*α* and REV‐ERB*β* in the secondary feedback loop [30]. The expression products of these genes include PER1, PER2, CRY1, CRY2, and several other peptides, such as casein kinase 1ε (CK1ε), which together form an inhibitory complex. When the activity of this inhibitory complex reaches a threshold level, it attenuates the transcriptional activity of CLOCK and BMAL1. Therefore, the level of the PER and CRY inhibitory complex decreases, thereby relieving the inhibition of CLOCK and BMAL1 activity. This allows transcription and translation of PER and CRY to begin a new 24‐h cycle, thus maintaining the circadian rhythm [31–38].

Circadian rhythm disorders arise not only from a dysfunction of the internal biological clock but also from its misalignment with the external world, as seen in shift work disorder, jet lag, and various factors of modern life such as artificial light at night. The pervasive use of modern artificial lighting impinges on the natural Circadian rhythm patterns. Exposure to artificial light disrupts the TTFL and cellular functions within the suprachiasmatic nucleus (SCN), affecting their timing and coherence. As the central circadian pacemaker, the SCN’s rhythmic and synchronized output is essential for orchestrating a wide array of physiological and behavioral processes. Circadian disruption and internal desynchrony occur when mistimed or conflicting synchronization signals distort the timing of the central SCN rhythm or those in peripheral tissues [39, 40].

Circadian rhythm disorders arise from disruptions to the body’s internal biological timekeeper, which governs the 24‐h cycle of numerous physiological processes. Key functions such as brain wave patterns, hormone secretion, and cellular repair are all intrinsically tied to this central clock [41]. Circadian rhythm disruptions precipitate metabolic abnormalities, immune dysregulation, and cognitive impairments, thereby contributing to a range of health conditions, including obesity, cardiovascular diseases, and sleep disorders [33, 42, 43]. Accumulating evidence reveals intricate and interconnected relationships among circadian rhythms, metabolic processes, and the immune system [44]. Notably, circadian rhythms modulate CD8^+^ T‐cell responses within the adaptive immune system, significantly influencing their activation and functional capacity [45, 46]. While inflammatory responses can disrupt circadian rhythms in peripheral tissues and innate immune cells [46], it is important to recognize that different immune cell types play distinct roles in inflammation [44]. Consequently, the effects of circadian immune metabolism are cell‐type‐specific, reflecting the complexity and temporal diversity of immune responses [44].

The impact of circadian rhythms on asthma manifestations is multifaceted, encompassing both symptomatic expressions like nocturnal coughing and dyspnea, and physiological parameters including airway hyperresponsiveness (AHR), airway resistance, mucus hypersecretion, and inflammatory processes [47, 48]. It is crucial to distinguish between diurnal variations—observable 24‐h fluctuations that can be influenced by external factors like sleep–wake cycles—and endogenous circadian rhythms, which are self‐sustaining oscillations generated by intrinsic molecular mechanisms independent of environmental cues.

Clinical observations consistently demonstrate a striking temporal pattern in asthma severity. Nocturnal and early morning hours represent a high‐risk period for asthma exacerbations and mortality across all age groups, with peak airway constriction typically occurring around 4 a.m. [49, 50]. This rhythmicity is reflected in various asthma indicators: fractional exhaled nitric oxide (FeNO) levels exhibit significant diurnal variation, with nadirs at 4 a.m. (25.5 ppb) compared to daytime peaks at 10 a.m. (37.5 ppb) [51, 52]. Similarly, AHR and exercise‐induced bronchoconstriction demonstrate distinct time‐dependent patterns, being most pronounced during the night [53, 54].

Epidemiological evidence further substantiates the circadian–asthma relationship. A cross‐sectional analysis of 11,475 U.S. adults revealed that late bedtimes (after 23:00) correlated with increased asthma prevalence [55]. More compellingly, UK Biobank data from over 280,000 participants demonstrated that long‐term night shift workers faced elevated asthma risk, with all shift workers showing higher odds of wheezing symptoms [54]. Critically, these pulmonary function rhythms persist under constant routine conditions, confirming they are governed by endogenous circadian systems rather than merely reflecting behavioral or environmental cycles [56].

Given the critical role of circadian‐immunometabolic crosstalk in asthma, it is imperative that we elucidate these mechanisms to guide the development of new interventions and improve treatment strategies.

## 2. Circadian Regulation of Asthma Immunity and Inflammation

In asthma, the immunological mechanism refers to the initial, antigen‐specific activation of the adaptive immune system. This is typically driven by the aberrant activation of T‐helper 2 (Th2) cells in response to allergens or other triggers. These cells release a specific set of cytokines (IL‐4, IL‐5, IL‐13), which in turn instruct B cells to produce allergen‐specificimmunoglobulin E (IgE). This sensitization phase represents the disease’s underlying “programing.” In contrast, the inflammatory response is the direct, downstream consequence of this immunological activation. Upon re‐exposure to the allergen, IgE binding triggers mast cell degranulation and the coordinated recruitment and activation of effector cells like eosinophils, basophils, and neutrophils. This results in the hallmark features of asthma: airway swelling, mucus hypersecretion, and bronchial hyperreactivity. Thus, the immunological mechanism initiates the disease state, while the inflammatory response executes the tissue damage and clinical symptoms.

### 2.1. Immunological Mechanism and Inflammatory Response of Asthma

#### 2.1.1. Immune Response

Asthma is intricately linked to allergen‐induced T2 immune responses, yet these reactions are shaped by environmental cues that can simultaneously activate innate and non‐T2 pathways, which may either aggravate or protect against disease [57].

The canonical T2 response is driven by CD4^+^ Th2 cells and group 2 innate lymphoid cells (ILC2s) that secrete IL‐4, IL‐5, and IL‐13, cytokines central to asthma pathobiology [58]. Consequently, adaptive immunity—via T–B cell collaboration—promotes IgE synthesis, airway hyper‐responsiveness (AHR), goblet‐cell metaplasia and eosinophilic inflammation, all hallmark features of T2‐high asthma [59]. Although Th2 cells were historically viewed as the dominant source of T2 cytokines, ILC2s have emerged as a potent innate counterpart; their numbers are expanded in blood and bronchoalveolar lavage of patients with severe T2 asthma, and they can sustain airway inflammation even when adaptive immunity is suppressed [60–63]. Importantly, ILC2s retain plasticity, enabling them to co‐produce IL‐17 or IFN‐γ under certain inflammatory cues, thereby bridging T2 and non‐T2 endotypes [61–63].

In contrast to the T2‐high endotype, non‐T2 asthma is largely dissociated from eosinophilic inflammation and is often characterized by neutrophilic or pauci‐granulocytic profiles. Its underlying mechanisms are diverse, involving multiple immune cell types (such as Th1 and Th17) and cytokines (including IL‐6 and IL‐17). Furthermore, the formation of neutrophil extracellular traps (NETs) and the activation of inflammasomes have been implicated in severe neutrophilic asthma [17, 64–66]; it exhibits blunted responsiveness to inhaled corticosteroids and involves innate immune modules—including ILC1/ILC3 subsets, bronchial epithelial cells, macrophages and dendritic cells—that respond to viral infections, pollutants or bacterial stimuli rather than to classical allergens [64–66].

#### 2.1.2. Nonimmune Response

The interplay between immunity and inflammation in asthma is intricate [57]. Asthma’s inflammatory profile encompasses the infiltration of inflammatory cells within the airways in response to triggers such as viruses, pollutants, and allergens [67]. Cytokines not only trigger immune responses within the adaptive and/or innate immune systems but also instigate a range of nonimmune reactions. These include oxidative stress, alterations in airway epithelial and smooth muscle cells, airway remodeling, as well as processes like apoptosis and autophagy. Oxidative stress in asthma primarily arises from an excessive generation of reactive oxygen species (ROS) and reactive nitrogen species (RNS) by inflammatory and structural cells, which overwhelms the endogenous antioxidant defenses. This imbalance promotes a state of heightened oxidative burden, leading to cellular damage, amplification of the inflammatory response, and direct injury to airway tissues [68, 69]. When these species are produced in excessive amounts or when the body’s antioxidant defenses are inadequate, they can exert harmful effects on airway cells and tissues [70, 71]. Allergic airway inflammation in mice is linked to heightened oxidative stress, predominantly affecting airway epithelial cells and macrophages. This condition is exacerbated by vitamin E depletion, which enhances sensitivity to formylcholine, a response that can be modestly attenuated by antioxidant supplementation [72]. Enhanced contractility of airway smooth muscle cells, a pivotal characteristic of asthma, is potentially associated with oxidative stress‐induced activation of RhoA and calcium Ca^2+^ channels [73]. Occupational allergens have the capacity to trigger disruptions in autophagy within lung epithelial cells, smooth muscle cells, and dendritic cells. These cellular dysfunctions can contribute to the development of occupational asthma (OA) through their involvement in inflammatory responses, oxidative stress, and cell death processes [74].

### 2.2. Circadian Characteristics of Asthma

A pivotal characteristic of asthma is the manifestation of symptoms in a circadian rhythmic pattern [75, 76]. Around three‐quarters of asthma patients report a notable exacerbation of symptoms or an increase in severity during the nighttime or early morning hours [56]. In patients with nocturnal asthma, the airways have been found to harbor elevated levels of inflammatory cytokines [77]. Several studies have indicated that the threshold dose of allergens required to provoke bronchospasm fluctuates with the time of day, demonstrating that allergens exert their most potent effects during the nocturnal hours [78, 79].

The cellular circadian system automatically modulates the interactive positive and negative transcription and translation feedback loop, which comprises at least nine key components: PER1, PER2, PER3, CLOCK, BMAL1, CRY1, CRY2, CK1ε, and TIM. These elements interact in a complex oscillatory network to regulate the diurnal rhythm of gene expression [80]. The core circadian proteins CLOCK and BMAL1 initiate the rhythmic transcription of the period (PER1, PER2, PER3) and cryptochrome (CRY1, CRY2) genes [34]. Subsequently, the resulting PER and CRY proteins assemble into complexes that translocate to the nucleus. Within the nucleus, CRY exerts a repressive effect on CLOCK and/or BMAL1 transcription, while PER modulates BMAL1 transcription. Additionally, the orphan nuclear receptors REV‐ERB*α* and ROR*α* play pivotal roles in the transcriptional regulation of BMAL1; REV‐ERB*α* acts as a repressor, and ROR*α* functions as an activator, both interacting through a shared response element. This intricate interplay fine‐tunes the circadian transcriptional machinery [52, 81, 82]. Circadian clock genes play a crucial role in regulating inflammatory responses and significantly contribute to pulmonary inflammation, fibrosis, immune reactions, and glucocorticoid signaling within the lungs [83–85]. Consequently, the body of evidence indicating the significance of circadian rhythms in the pathogenesis of asthma is mounting.

## 3. Effects of Circadian Rhythm on Lung Function in Asthma

### 3.1. Effects of Circadian Rhythms on Lung Physiology and Pathology

Circadian rhythms shape two key facets of asthma pathophysiology: airway caliber and sensitivity to inhaled allergens. Bronchial smooth‐muscle tone peaks between 2 a.m. and 5 a.m., producing the greatest airway narrowing and the highest likelihood of nocturnal symptoms [75, 86]. Parallel circadian swings in mast‐cell reactivity amplify allergen‐induced bronchospasm during the same overnight window, with the most acute responses recorded at midnight and in the early morning hours [78, 87].

Lung function undergoes natural fluctuations throughout the day, intricately linked to the body’s circadian rhythm. Typically, in individuals with optimal health, pulmonary capacity reaches its zenith around 4 p.m., subsequently waning to its nadir around 4:00 a.m. [88, 89]. Disturbances in the circadian rhythms are increasingly recognized as pivotal contributors to the pathogenesis of chronic lung diseases [90]. Emerging research underscores the pivotal role of circadian rhythm dysregulation in the pathophysiology of chronic pulmonary diseases, manifesting through the exacerbation of oxidative stress, the propagation of inflammatory cascades, perturbations in metabolic homeostasis, oscillations in oxygenation status, augmented mucus hypersecretion, impaired autophagic flux, and the derangement of pulmonary function [91].

### 3.2. Circadian Rhythms Influence the Activation of Immune Cells and the Release of Inflammatory Mediators in Lung Tissue

Researchers have identified notable disparities in the expression of nine key circadian clock genes between individuals with asthma and those without the condition, as well as between asthmatics who experience nocturnal symptoms and those who do not [92].

BMAL1, a pivotal component among circadian clock genes and proteins, exerts anti‐inflammatory effects. The targeted ablation of this solitary clock gene leads to the abolition of all rhythmic behavioral patterns [93]. The discovery that BMAL1 serves as a potent negative regulator in myeloid cells, particularly in allergic asthma, coupled with the observation that its expression dips during nighttime, may elucidate the nocturnal exacerbation of asthmatic symptoms [94]. The suppression of BMAL1 by PM2.5 exposure not only influences p53 protein levels but also modulates the autophagy response, which can directly affect airway epithelial cell function and contribute to the structural changes seen in airway remodeling in asthma [95]. In murine asthma models, the deficiency of BMAL1 is correlated with heightened airway inflammation and augmented airway resistance. This deficiency elicits pronounced inflammatory responses in pulmonary tissue, characterized by elevated eosinophil counts and increased levels of IL‐5, consequently leading to exacerbated asthma symptoms [94, 96]. In the ovalbumin (OVA)‐induced asthmatic murine model, the expression of the circadian rhythm‐associated gene BMAL1 orchestrates the periodic modulation of Mucin (MUC) 1 within the airway and pulmonary tissues, as delineated by a study involving a comprehensive analysis of circadian gene expression patterns. This study highlights the complex interaction between the circadian clock and the pathophysiological features of asthma, particularly highlighting the role of BMAL1 in the regulation of MUC1 expression, which is a key component in the airway’s mucosal defense mechanism. The findings suggest that the circadian regulation of MUC1 by BMAL1 may hold significant implications for understanding the temporal dynamics of asthma symptoms and potentially inform therapeutic strategies targeting the circadian clock to mitigate asthma pathologies [82]. In BMAL1‐deficient mice, circadian fluctuations in active inflammatory cytokines are abolished or suppressed by drugs that regulate circadian proteins [97, 98].

YTHDF1, identified as a significant modulator of airway inflammation in asthma, augments the translation of CLOCK in an m6A‐dependent manner. This enhancement of CLOCK translation by YTHDF1 is instrumental in the inflammatory process, as YTHDF1 further activates the NLRP3 inflammasome, leading to the production of IL‐1*β*, a key cytokine in asthma‐related airway inflammation. However, the deletion of CLOCK abrogates these inflammatory phenotypes, indicating the integral role of CLOCK in the YTHDF1‐mediated inflammatory response in asthma [99].

Proteins from the PER family exert a significant influence on lipid metabolism [100–102], play a crucial role in modulating various biological processes that are implicated in asthma [103–105]. Research has demonstrated that CRY genes influence the onset and progression of allergic conditions, including asthma, by modulating the integrity of the airway epithelial barrier. This suggests that the regulation and stabilization of CRY genes could potentially mitigate asthma symptoms triggered by allergens, such as dust mites [106].

Furthermore, an additional negative feedback loop, exerting direct influence on the core circadian mechanism, is constituted by REV‐ERB*α*/*β* (nuclear receptor subfamily 1/2) and retinoic acid receptor‐related orphan receptors (RORs). These factors govern the expression of the activator BMAL1. Collectively, these interlocking feedback loops, known as TTFL, function in concert to engender the circadian rhythm, fine‐tuning the intricate balance of the biological clock [107, 108]. REV‐ERB*α* can suppress the activation of IL‐6 expression by binding to the NF‐*κ*B complex, specifically the RelA/p65 subunit, thereby modulating NF‐*κ*B‐driven transcription. The transcription factor activator protein 1 (AP‐1) and NF‐*κ*B have distinct sequence motifs that overlap with the consensus binding sequence of the REV‐ERB*α* promoter. This overlap suggests a significant role for REV‐ERB*α* in regulating oxidative stress and inflammation, highlighting its potential as a key modulator in these pathways [109, 110]. Exposure to Th2 cytokines or house dust mite (HDM) extract notably decreased cellular impedance, as illustrated by the diminished transepithelial electrical resistance (TEER) measurements reported by Duraisamy SK et al. Conversely, preemptive treatment with the REV‐ERB*α* agonist SR10067 significantly alleviated the barrier dysfunction triggered by Th2 cytokines. This intervention resulted in reduced permeability and enhanced localization of TEER, along with the improved distribution of adherens junction (AJC) and tight junction (TJ) proteins. Furthermore, it effectively regulated the mRNA and protein expression levels of circadian genes CLOCK, BMAL1, CRY1, PER1, PER2, Nr1d1, Nr1d2, and Nfil3. This discovery reveals, for the first time, the role of REV‐ERB*α* activation in modulating the epithelial barrier function [111]. In vivo and ex vivo examinations of precise mouse lung sections revealed significantly varied AHR response times, peaking at dusk during the onset of the active phase. These studies demonstrated that the circadian influence on asthmatic responses was eliminated in mice deficient in the clock gene REV‐ERB*α*, underscoring the gene’s pivotal role in mediating daily fluctuations in asthma susceptibility [52].

## 4. Natural Drug Ingredients Regulate Circadian Rhythms to Treat Asthma

Treatments for type II‐high asthma include glucocorticoids combined with bronchodilators and monoclonal antibodies. However, some severe patients still find it difficult to effectively control their symptoms, with recurrent attacks occurring over a long period of time [112]. Due to the potential side effects of long‐term and high‐dose glucocorticoid use, it is necessary to seek more personalized and precise treatment plans, tailoring them to different types of asthma patients to maximize the therapeutic effect and reduce the risk of adverse reactions. Traditional Chinese medicine (TCM) represents a rich repository of natural compounds with significant potential for circadian rhythm modulation, offering a novel therapeutic avenue for chronic inflammatory respiratory diseases such as asthma. A growing body of evidence suggests that the therapeutic benefits of many TCM preparations may stem from their ability to recalibrate disrupted biological clocks, thereby restoring homeostasis and mitigating disease‐specific pathologies [113].

The mechanistic link between circadian modulation and asthma therapy is exemplified by specific TCM‐derived compounds. Bavachalcone, from *Psoralea corylifolia L*., functions as a ROR*α* agonist, enhancing the expression and oscillation of core clock genes like BMAL1 [114–116]. Given that ROR*α* plays a key role in regulating inflammatory pathways, the activation of this pathway by bavachalcone holds direct therapeutic significance for asthma. By reinforcing circadian rhythmicity, it may help suppress the aberrant innate immune activation and production of pro‐inflammatory cytokines (e.g., IL‐1*α*) that characterize chronic airway inflammation, thereby potentially reducing cellular senescence and tissue damage in the lungs.

**Figure 1:**
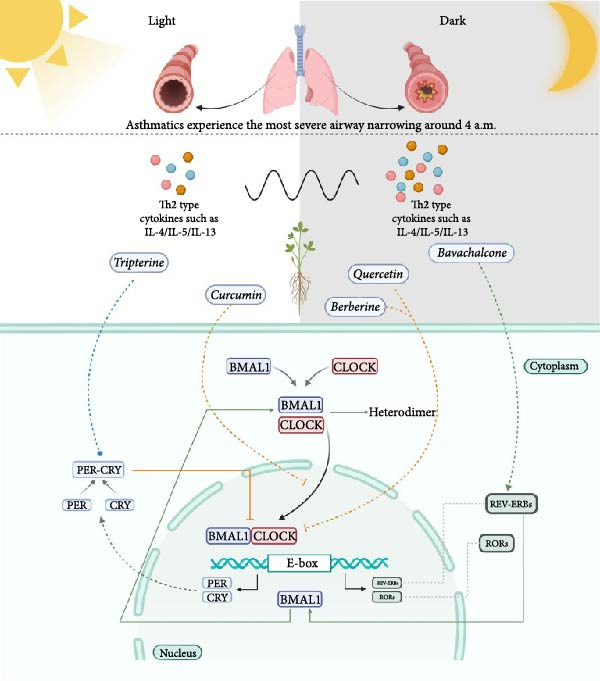
Circadian rhythm mechanisms and the potential targets of natural medicines and their components in regulating lung circadian clock: Lung function naturally fluctuates throughout the day. Asthma patients experience the most severe airway narrowing around 4 a.m., and the fluctuations of Th2‐type cytokines such as IL‐4/IL‐5/IL‐13 reach their peak. BMAL1 and CLOCK combine to create a heterodimer, which then enters the cell nucleus and triggers the transcription of numerous genes, encompassing the circadian‐associated genes PER, CRY, REV‐ERBs, and RORs. Natural medicines and their components, such as bavachalcone, quercetin, berberine, tripterine, and curcumin, have corresponding activating or inhibitory effects on REV‐ERBs, PER‐CRY, and BMAL1/CLOCK, respectively. Thus, they can regulate the lung circadian clock and improve chronic airway inflammation.

Berberine (BBR), a principal isoquinoline alkaloid derived from Coptidis rhizoma [117], exerts a significant inhibitory effect on the activity and expression of BMAL1 and NLRP3 in a concentration‐dependent manner. This inhibition suggests that BBR functions as an agonist for REV‐ERB*α*, modulating the circadian clock and inflammation‐related pathways [118]. The inhibition of NLRP3, a critical component of the innate immune system known to drive steroid‐resistant neutrophilic inflammation in severe asthma, positions BBR as a potential agent for managing difficult‐to‐treat asthma phenotypes. Its circadian‐targeted action helps synchronize the inflammatory response, preventing its overactivation [119]. Furthermore, traditional medicinal herbs such as *Artemisia argyi*, *Polygala tenuifolia*, *Pueraria lobata*, *Platycodon grandiflorus*, *Chrysanthemum morifolium*, *Paeonia lactiflora* (also known as schirmiae peony), cicada exuviae, *Perilla frutescens* leaf, *Syzygium aromaticum* (clove), *Uncaria rhynchophylla*, *Lonicera japonica*, *Alpinia galanga* (galangal), *Perilla frutescens* seed, *Acorus calamus* (calamus root), *Prunus mume* (dark plum), and *Selaginella doederleinii* (scalene) have the potential to modulate circadian rhythms in U2OS cells stably expressing the BMAL1‐dLuc reporter (Figure 1) [113].

Numerous drugs used in treating inflammatory and metabolic diseases directly target genes with circadian rhythms, highlighting the therapeutic potential of chronobiologically informed drug targeting [120]. Drugs that target the stabilization of CRY proteins have been found to possess anti‐inflammatory properties, which are beneficial in the context of inflammatory conditions [121]. Furthermore, the modulation of REV‐ERB, a nuclear receptor that regulates transcription, has shown potential in mitigating both atherosclerosis and cancer, highlighting its broad therapeutic implications [122, 123].

In conclusion, while preclinical findings suggest therapeutic promise for TCM‐derived compounds in fine‐tuning circadian gene expression within the lung and immune cells, this potential remains largely hypothetical in the context of asthma. The current evidence base, derived primarily from in vitro studies and models of unrelated diseases, necessitates a critical and cautious interpretation. To translate this chronobiological approach into viable asthma therapeutics, future research must first address fundamental gaps. This includes elucidating the precise mechanisms of action in relevant pulmonary and immune cell types, establishing robust pharmacokinetic and safety profiles, and ultimately validating efficacy through well‐designed randomized controlled trials in asthma populations.

## 5. Discussion

Asthma, a chronic inflammatory airway disease, is notably influenced by circadian rhythms. Symptoms like coughing, wheezing, and shortness of breath tend to intensify during nighttime and early morning periods. This phenomenon is not only related to increased airway resistance and hyperresponsiveness but also associated with immune metabolic disorders and the role of core circadian genes. Circadian rhythms regulate multiple biological processes in the body through TTFLs. Key genes such as BMAL1 and CLOCK play important roles in regulating gene expression, metabolism, and immune responses. Studies have shown that circadian rhythm disorders can lead to metabolic abnormalities, immune dysregulation, and worsening of asthma symptoms. BMAL1 and CLOCK regulate inflammatory responses, airway smooth muscle function, and oxidative stress. The circadian activities of eosinophils and neutrophils significantly contribute to asthmatic inflammation. This process is partly governed by core circadian clock genes, such as CRY and REV‐ERB*α*, which participate in asthma pathogenesis by regulating airway epithelial barrier function and inflammatory responses.

Altering circadian rhythms presents novel avenues for managing asthma. The regulation of circadian rhythms is vital for immune responses, airway inflammation, and the rhythmic patterns of lung function in asthma, positioning it as a potential therapeutic target. Natural medicines, such as psoralen and BBR, have been found to restore circadian rhythm balance by modulating the expression of BMAL1 and REV‐ERB*α*, thereby alleviating asthma inflammation. Medications that stabilize CRY protein or activate REV‐ERB are thought to hold promise for diminishing airway inflammation and oxidative stress. Targeting the regulation of clock genes holds promise for innovative asthma treatments, especially for patients with severe nighttime symptoms.

Therefore, circadian rhythms significantly impact the pathophysiological processes of asthma. They are not only a major determinant of the temporal distribution of asthma symptoms but also a potential new research direction for asthma onset and treatment. In‐depth studies of their molecular mechanisms and gene regulation may bring breakthroughs in asthma treatment. The development of natural medicines and targeted intervention of circadian rhythm genes could become an important strategy for future precision treatment of asthma.

## Ethics Statement

The authors have nothing to report.

## Consent

The authors have nothing to report.

## Conflicts of Interest

The authors declare no conflicts of interest.

## Author Contributions

**Zheng Xue, Yazun Liu, and Wenbo Yao**: writing – review and editing, conceptualization, methodology, project administration. **Feiyan Cheng**: writing – review and editing, conceptualization, methodology. **Manchen Lu**: writing – original draft, conceptualization, methodology. **Xiaoman Zhao**: writing – review and editing, formal analysis. **Yuanyuan Zhang, Jing Chen, Shengyuan Huang, and Xiaoman Zhao**: writing – review and editing. Manchen Lu and Feiyan Cheng contributed equally to this work.

## Funding

This work was supported by the National Natural Science Foundation of China (Grants 82274576 and 82205179).

## Data Availability

The data that support the findings of this study are available from the corresponding author upon reasonable request.
