# Correction to “The Circadian Rhythm of Asthma Immune Metabolism”

**DOI:** 10.1155/jimr/9801216

**Published:** 2026-04-21

M. Lu, F. Cheng, X. Zhao, et al., “The Circadian Rhythm of Asthma Immune Metabolism,” *Journal of Immunology Research*, 2025, 3246881, https://doi.org/10.1155/jimr/3246881.

In the article, there was an error in the order of the authors, which has been corrected from:

• M. Lu, F. Cheng, X. Zhao, Z. Wang, J. Chen, Y. Zhang, S. Huang, X. Zhang, Z. Xue, Y. Liu, W. Yao

to:

• M. Lu, F. Cheng, X. Zhao, Z. Wang, J. Chen, Y. Zhang, S. Huang, X. Zhang, Y. Liu, W. Yao, Z. Xue

All authors agree to the revised author order.

We apologize for this error.

